# Supplementary material for: From cognitive control to visual incongruity: Conflict detection in surrealistic images
Source: PLoS One. 2020 Jun 4;15(6):e0224053. doi: 10.1371/journal.pone.0224053 (PMC7272058; doi:10.1371/journal.pone.0224053)
Supplement: Attachment — Submitted filename: Ruzzolietal_Response2Rev.docx [file pone.0224053.s001.docx]

**Reviewer #1: The paper describes a potentially sound research question, exploring the brain correlates and the memory processing associated to the vision of incongruous images in the advertisement domain. Even if this research question could be particularly intriguing, the work has too many theoretical and methodological flaws to be considered publishable in Plos One in my opinion. In the following, major and minor criticisms to the paper are listed:

INTRODUCTION
- The introduction is only drafted: while the aim of the paper can be grasped, neither a theoretical proposition, not specific hypotheses have been described by the authors. The pre-registration of the work is a valuable add to a paper, but the published paper must be a self-standing work; it is indeed necessary that the text contains all information necessary to the reader to understand the theoretical propositions, the aims, the originality, and the hypotheses of the work. At the moment, none of these points is adequately described in the introduction.**

**RESPONSE 1**: We thank the Reviewer for this comment, and we apologize for the lack of clarity. It is important to mention that a theoretical grounding as well as a clear hypothesis, back the present study. In fact, pre-registering the project helps one to have a clear hypothesis. We apologize for not making it clear in the paper. The introduction has been rewritten to make the theoretical framework and the hypotheses clearer, without need to resort to the pre-registration document.

We want to point out that the theoretical framework we adopted in planning our study and interpreting the results is the *conflict monitoring theory* (Botvinick *et al.*, 2001; Shenhav *et al.*, 2013, 2016; Inzlicht *et al.*, 2017), which is a popular theory in the domain of cognitive control. In the present version of the manuscript, the theoretical framework is made clear from the opening statement.

The originality of our study, however, was to bring this cognitive framework outside the Lab and ask if it can be applied to a more realistic context, such as advertising. We had specific hypotheses in mind, which oriented us in stimuli selection and in the analysis pipeline (as it is described in the Method section).

Based on the mentioned framework of the cognitive monitoring theory we predicted that images contaning visual/semantic incongruity would be associated with higher Theta power at fronto-central sensors, compared to control images, analogous to what happens in more classical laboratory protocols of conflict such as the Stroop task (Stroop, 1935). Following this prediction, we also hypothesized that the incongruent images would be remembered better.

**Moreover, while the pre-registered hypotheses were simple and clear, in the present paper the theoretical and methodological questions seem to shift from incongruity to surrealism, which are distinct concepts. The research questions seem therefore to be different from the questions described in the pre-registered work.**

**RESPONSE 2**: We apologize for the lack of conceptual clarity in the introduction. We agree with the Reviewer that incongruity and surrealism are not the same concept, nor that our original intention was to test the brain response to surrealism in general. Our initial hypothesis was about a special form of visual incongruity (see Response 1, above), often used as creative technique in advertisement, which uses surrealistic images to deliberately defy the message by the juxtaposition of elements taken from un-matching context. In the present version of the manuscript, we only refer to surrealism in general terms in the introduction and discussion, while incongruity is used to operationalize the experimental question. Also, we hope that the relationship between the operationalization of the question and surrealism has been put in sufficient perspective.

**- The authors wrote "In the present study, we tested the hypothesis that visual imagery that contains incongruent meanings, such as that used in advertising,..." This sentence is not clear, representing a generalization of the use of visual imagery in advertising.**

**RESPONSE 3**: We thank the Reviewer for this comment. We changed this sentence to avoid the confusion (page 3): "*In the present study, we tested the hypothesis that images that juxtapose incongruent meanings, such as ads exploiting surrealistic techniques, will produce conflict adjustments similar to those in classical conflict protocols.*"

**- The authors wrote: "Conflict response will be reflected in frontal-medial Theta power (4-8Hz), compared to control images that do not contain incongruity." Please reformulate: the two terms of the contrast are posed on different levels (conflict response vs control images)**

**RESPONSE 4**: Thanks, the introduction has been rewritten, and this sentence has been removed.

**- The authors wrote: "Furthermore, we assess the impact of conflict on subsequent memory by introducing a recall task one week after exposure to the images. We address the specific hypothesis regarding the memorability of surrealist imagery, implicit in advertising. Cognitive neuroscience has suggested a relationship between visual memory capacity and semantic consistency (see Brady, Konkle, & Alvarez, 2011), whereby objects that are inconsistent with the surrounding scene are remembered more (see also Friedman, 1979; Krebs, Boehler, De Belder, & Egner, 2015). The impact of surrealistic ads on memory has been only tested in a seminal study by Homer & Kahle (1986) using a free-recall test. They found that participants who saw surrealistic ads were less inclined to errors, compared to a control group, thus supporting the proposal that surrealistic images strengthen memory." Please be more precise and specific:
- "after exposure to the images" which kind of images?
- imagery or images? Surrealist imagery and surrealist images are two different concepts.**

**RESPONSE 5**: Thanks, the sentence has been changed (page 3): "*Furthermore, in the present study, we assessed the impact of conflict on subsequent memory by introducing a recognition task one week after the first exposure to both the incongruent and control images during the EEG test"*

The memory test was performed one week after the EEG test, in which both the incongruent and the control images were presented to the participants. As explained in the Methods, during the EEG test (that was the first exposure to the images), participants were not aware that they would have been asked to recall them one week after.

We thank the Reviewer also for drawing our attention towards some very important linguistic oversights (e.g., images vs imagery), which we corrected throughout the manuscript.

**- Please be more specific on the main features and distinctive elements defining surrealistic ads. This is a critical point of the paper, which never defines the main constituents of surrealistic images, surrealistic imagery, and surrealistic ads (which are three different concepts and research fields). Moreover, a raw generalization from surrealism to incongruity has been made by the authors, which is a high level simplification of the concept of surrealism.**

**RESPONSE 6**: The Reviewer is correct that we did not carefully describe what we meant by surrealistic images and we also used terminology imprecisely. In the present version of the manuscript, we have been more careful in these aspects.

First, we have employed the term images (vs. imagery), as it seems to be the most adequate. Second, when ads make use of surrealistic images as a creative technique, we do still use the term surrealistic ad; however, the term incongruent is used most of the time for operationalization (also, see our Response 2, above).

Regarding the main feature of the surrealistic ads/images. We hope we have already clarified this question, at least partly, in our previous responses. Basically, surrealistic images (and ads that employ these images) resort to several creative techniques, including representing conflicting meanings. We narrowed down into these type of surrealistic images for an operational definition of our research question, and hypothesis. Hence, we selected real ads that presented incongruent visual (consequently semantic) meaning only by the images (not in connection to the brands, iconic symbols, or through text). One initial selection was searching for 'Creative Advertising', 'Smart Ads', 'Innovative Advertising', and 'Surrealist Ads', with no other specific criteria in mind. A precise description of the criteria and procedure used for image selection can be found in pages 4-5 of the manuscript.

**- The authors defines conflict as a creative device; however, it is hard to define "conflict" as a creative device, whereas it is usually used as a creative technique or as an ideative method.**

**RESPONSE 7**: Thanks, the sentence has been changed and the controversial definition has been removed. Also, in other instances, we used the term "creative technique".

**METHOD
- The method used to operationalize the construct of incongruity is essentially based on wrong assumptions, since it used a methodology to define and measure a different construct: divergence. The authors wrote: "All 240 images initially selected with the methods above were then subjected to a survey to validate the levels of visual incongruity for each image by independent judges. The survey consisted of determining the degree of agreement of two statements regarding the categories of originality and synthesis from Smith et al.' study (Smith et al., 2007), which can be considered an objective way to determine image incongruity. " The two categories used by Smith et al. (2007) are only two of a series of categories defining the concept of divergence (applied to the specific case of advertising). All these categories were extracted from classical definition of divergent thinking (using the criteria by Guilford, Torrance, etc.). Incongruity and divergence (especially if referred to divergent thinking) are, from a theoretical, practical, and methodological points of view, distinct and different psychological constructs. The fact that they can overlap requires a specific scientific analysis, which is not performed in the present work. All in all, the choice of a method used to measure a construct to define a different construct is scientifically and technically wrong.**

**RESPONSE 8**: We thank the Reviewer for this important point. Cognitive conflict is described as "the simultaneous activation of incompatible and competing representations" by the *conflict monitoring theory* (Botvinick *et al.*, 2001; Shenhav *et al.*, 2013, 2016; Inzlicht *et al.*, 2017). Bridging from this conceptual definition to more practical, real-life terms, especially those outside the prototypical laboratory tasks often used within the cited theory, is challenging. We used Smith et al. (2007) work as point of reference and inspiration because it is specifically about the determinants of ad creativity, which was the base of our experimental materials. As the reviewer rightly points out, the authors of that work define creativity in advertising as divergence, and in particular, as '*the extent to which an ad contains elements that are different, novel, unusual or original*'. Smith et al. (2007) also developed and validated scales for measuring the key components of ad creativity over a series of six pre-tests involving 1,250 respondents. It was not the purpose of our work to adapt or establish an operationalization of conflict with the same precision, or else to assume that one (creativity) equates the other (congruence). Instead, we sought for some grounding of our characterization of the stimuli. To do so, we focused on two of the seven main indicators of ad divergence originally considered by Smith et al. (2007), namely the categories of *originality* and *synthesis*, as the ones closer to the definition of cognitive conflict that has been traditionally given in the mentioned cognitive theory*.*

We agree with the Reviewer that measuring a construct (divergence) to define another construct (congruency) is not optimal. We do discuss this in the current version of the manuscript (see p. 5).

We would like to bring to the Reviewer's attention that we are, as far as we know, on uncharted territory. To the best of our knowledge, no one has bridged the conceptual gap between the concept of cognitive conflict in the theory and cognitive conflict as it could happen in real-life domains, such as advertisement or art that could be defined as surrealist. We felt uncomfortable by making unwarranted assumptions about what conflict is when taking the concept out of the confines of the cognitive control theory, and therefore resorted to the strategy to find in the existing literature on creativity the subset of features that, to our interpretation, is related to conflict (i.e., the construct of divergence). Hence, our focus, was not to rate different images along the many faces creativity can take. Instead, our focus was to estimate incongruity using the two dimensions among the original seven that define divergence. A clear definition of conflict, outside idealized laboratory protocols, is still awaiting and far beyond the scope of our empirical research. We agree that we did not optimally develop a theoretical construct, which was not our intention; instead, we ran the survey deeming to try and ground the assumptions underlying our hypothesis and predictions more solidly than it would have been the case without the survey. Our empirical results seemed to justify the assumption, although this is admittedly, an *ex-postfacto* argument.

We have made these considerations in the manuscript (pages 5 and 12).

**- Moreover, since perception represents a critical key in the processing of congruency/uncongruency images must be controlled for their physical properties (at least high level processes).**

**RESPONSE 9**: This is an interesting point. As stated in the Method section, the control images were selected based on 'Google Reverse Image Search' and the website 'Yandex' (<https://yandex.com/>). We therefore provided the system with the incongruent images, the database returned images similar for content, color, main element in the scene among which we choose the controls. We agree with the Reviewer that working with natural existing images, compared to laboratory stimuli (such as Gabors), lessens the degree of control the experimenter can exert upon stimuli. However, we do not think this can be a criterion to invalidate the study completely. In addition, whilst lessening control one can increase ecological validity. In the present version of the manuscript, we explicitly comment on this limitation for the reader's consideration (page 4).

**RESULTS
- Some imprecisions in reporting and interpreting the findings are present in the results section, which then engender a series of misunderstanding and wrong assumptions in the final discussion. The authors wrote: "Multiple comparison correction following Guthrie & Buchwald (1991) determined a threshold of 7 consecutive significant (p< 0.05) points (φ ~= 0.99, number of time points = 38, N=27, and Theta = .05). We found significant differences in frontal-medial Theta power in a 340 ms time window (between 370 and 710 ms), with 18 consecutive significant points, revealing that looking at incongruent images was associated to a higher Theta power compared to controls (see Figure 2)." It is here highly important to introduce and comment the meaning of this result. The difference is not an overall difference, but it is related to the time (and expecially to a specific time window). This is a fundamental distinguo, which is at the core of the emerging results.**

**RESPONSE 10:** We apologize for the lack of clarity about this point and have modified the manuscript accordingly. The EEG power in a given frequency can be analyzed in a window of interest by averaging the power measure across time, and then compare it between conditions (this is what we did in the first analysis). Another way to test EEG power effects, is to calculate the power evolution along the window of interest to find the time window significantly different between conditions, controlling for the autocorrelation in the signal (by Guthrie & Buchwald' correction, for example). This second approach is what we did in the second analysis. Using one approach or the other is a matter of prior expectations and analysis approach. Both are very common, not only the conflict/incongruency field, but in any instance of EEG of MEG analysis. In fact, beyond EEG and MEG, differences in brain activity must happen (assuming their existence) in a specific time window. Assuming that our reference to the second analysis as "time evolution" has been the origin of the misunderstanding, we add a specification on this point (page 10).

**- Correlations must be read/interpreted/discussed on the basis of the effect size and not on the basis of the statistical significance. Looking a the correlations on items and on individuals, the size of the effect is almost twice in the latter case (and negative) than in the former case.**

**RESPONSE 11**: First, we would like to apologize because there was a mistake in the reported confidence intervals of the item-by-item correlation now corrected in the main manuscript, this mistake does not change the significance or direction of the effect.

We agree with the Reviewer that the effect size of correlation is relevant in its interpretation and we mentioned it in the main manuscript (page 12). Nonetheless, we disagree with the affirmation that the correlation on individuals is almost twice than in the latter case (or negative). In the item correlation, the 95% confidence interval is [0.015 0.29], therefore our data supports that the effect is positive as it excludes the r=0 value, albeit it may vary from a very modest size effect of 0.015, to 0.29 (and is discussed as such in the paper). On the other hand, in the individual correlation, the confidence interval is [-0.65 0.03], which includes r=0, thus indicating that we cannot draw a conclusion on the individual correlation (not even regarding the direction of the effect or its size). Affirming that the latter effect size is double than the former would be an unfortunate interpretation of the point estimates disregarding the variability associated to the measures.

**DISCUSSION
- Starting from the first sentences of the discussion section it is still not clear the distinction between incongruent and surrealistic images. Are they the same? Why? Why not defining the images simply as incongruent as made in the registered protocol?**

**RESPONSE 12**: We apologize again for the lack of clarity. As in the Introduction, we also revised the discussion and removed from it many references to surrealism in general, as per our responses to the earlier points. We agree with the Reviewer that this point generated confusion, so we have used the concept of incongruency to operationalize the study and research hypothesis, and only refer to surrealistic images as the emblematic example we put at test and in the conceptual hypothesis. Please, consider, however, that we made an effort to select images that can be considered surrealistic in the study (see Method Section), this is why we feel we can speculate in this sense.

**- The authors wrote: "As predicted, albeit with a non-registered analysis pipeline, we found that surrealistic advertising imagery not only generated stronger Theta activity at frontal-medial scalp locations compared to control images, it also produced higher recognition rates in a memory task delivered one week after the exposure, a key behavioral metric of advertising effectiveness (Keller, 1987). Furthermore, item-by-item Theta power and subsequent memory recognition for the corresponding image showed a correlation. This suggests that the stronger the Theta response an image elicits in the brain, the more likely it will be remembered, albeit the effect size of this correlation was small." Results must be reported as they are. Authors find a difference in Theta only in relation to temporal dynamics and not as an overall effect; this point is critical and must be discussed. Moreover, the correlations showed that the variables are really weakly correlated, which is exactly the contrary of what authors expected and are sustaining (in the case of individual correlations the variables are negatively associated - with a stronger effect size than the effect discussed by the authors).**

**RESPONSE 13:** In relation to the temporal dynamic of the results, we refer the Reviewer to our previous response to Response 10, in which we hope to have clarified the time issue. Furthermore, we are indeed very cautious interpreting the weakly significant correlation, both in the results and in the discussion (but see also our previous Response 11). Therefore, we must disagree with the Reviewer because we were extremely careful in reporting the results as there are and, very important, we reported all the analysis and results we performed regardless of significance. This clarity might have generated some misunderstandings that we carefully tried to avoid rewriting some sentences in the description of the results.

**- The authors wrote: "Our results provide a viable experimental procedure and proof-of-concept data that helps bridge the gap between the scientific and creative approaches and uncover new insights into the relationship between cognitive conflict and creativity." It is totally unclear why it should give insight on this relationship. Creativity has not been explored in the present study.**

**RESPONSE 14:** We agree with the Reviewer. We hope we have clarified now the approach and intention of this study in Response 8. With respect to this particular sentence, we have changed it accordingly (page 11): "*Our results provide a viable experimental procedure and proof-of-concept data that helps bridge the gap between a scientific framework based on cognitive neuroscience and advertising, uncovering new insights into the relationship between cognitive conflict and incongruent images such as surrealistic ads."*

**- The authors wrote: "Therefore, extending the proposal by Inzlicht, et al. (2017), the creative communication strategy typically used in advertisement and arts not only elicits cognitive conflict and increased memory, as our data suggest, but might also be seen as an intrinsic reward, potentially linked to positive subjective experience (Chetverikov & Kristjánsson, 2016; Van de Cruys & Wagemans, 2011)." This is pure speculation which can not directly derived from the present results; ad hoc experimental studies should be made based on these hypotheses.**

**RESPONSE 15:** We agree, we indeed declare this to be a speculative claim. We would like to keep this speculation for the reader's consideration. The sentence has been re-nuanced, though (page 12).

**What do you mean for "creative communication strategy"? The (logical, conceptual, operational) gap between the images used in this study and a typical creative communication strategy is, to say the last, huge.**

**RESPONSE 16:** We hope to have clarified our position regards the images used, the selection criteria, and the theoretical framework of the present study in the previous responses and in the revised version of the manuscript. Sentences in which the definition was reported have also been re-formulated.

**Reviewer #2: By means of surrealistic images (semantically conflicting) presentation, this study has two main aims. The first one is to confirm the increase in theta oscillatory activity (4-8 Hz) over mid-frontal cortex; the second one is demonstrate, for the firs time, the neural basis of the increase memorability of the surrealistic semantic conflicting images. The results confirm the two hypothesis showing that observing incongruent images was associated to a higher theta power compared to controls and that viewing incongruent images also produced higher recognition rates compared to control images. The study is performed accurately and the results are interesting. However, in order to improve the quality of the paper and to deeper understand the results, I suggest to perform a new analysis comparing theta and alpha power in the recording sites.**

**My first methodological concern is about the category of incongruent images that is a bit vast and, for what I understood, it comprising a mix of images including art, advertising, objects, human and non human action, etc. Also the control images seem to be rather mixed. A categorization of the images belonging to control and experimental conditions and a precise matching between each category in both conditions should be given.**

**RESPONSE 17:** We thank the Reviewer for this comment, we have defined image selection criteria and the way we matched incongruent and control images more precisely in this new version of the manuscript (see pages 4-5).

Because we did not have a theoretical or empirical reason to be category-selective, we used different image categories to avoid constraining ourselves too much as to compromise the congruence manipulation. The reasons (a priori) to be more categorically selective would be to (1) investigate how the incongruency effect varies across categories, and/or (2) to reduce overall variability in our measurements. We did not intend to do (1), since we wanted to just proof the concept of cognitive conflict in surrealistic images, and we had no theoretical reason to investigate different categories. If something, the type of incongruity we were interested in (potential source of cognitive conflict) should be "category independent". Variability, in the case of (2), works against us, and therefore it was a risk of the experiment and not a systematic bias favouring our hypothesis. That is, if anything, this mix in image selection should have obscured potential effects rather than artificially heightening them.

**The survey performed in order to measure the degree of congruency/incongruency has two questions but it is not explained which one has been used for the images selection. Please, provide this information together with the two average scores (one for each question) of the 100 incongruent and 100 congruent images used as final experimental image set. Ideally the same survey should be done also on the filler images in order to exclude a bias of these images for congruency or incongruency scores.**

**RESPONSE 18:** Thank the Reviewer for this important point. Image selection has been described more precisely in this new version of the manuscript and the results from the survey have been summarized in the text (page 5).

**About the Theta and Alpha power I strongly suggest to perform a 2X2X2 Anova with the main factor of Condition (congruent and incongruent), Rhythm (theta and alpha) and Location (fronto-central cluster and parietal-occipital cluster). This analysis allows having more realistic overview of the brain responses during the observation of the two categories of images. In particular, it could allow to demonstrate that the increase of theta power is specific for fronto-central brain region.**

**RESPONSE 19:** We thank the Reviewer for this suggestion, nonetheless we do not agree that a 2x2x2 ANOVA analysis would be clarificatory (at least, of the specific questions under study). We explain the reasoning in detail below. Yet, since this analysis is nevertheless doable from a purely technical point of view, we have performed it for the Reviewer's request and show the outcome after the explanation.

First, this ANOVA compares / mixes different frequency bands of oscillatory activity (Alpha and Theta) for which we did not have any expectation or interest in comparing. Comparing different bands directly is not only futile in terms of our question, but in general, seems to be a strange comparison to make in oscillatory analysis unless some adjustments were made (e.g., what does it entail that power is different or equal in two different frequency bands? Different frequency bands in an EEG recording usually contain different amount of power under the same conditions).

Furthermore, in a 2x2x2 ANOVA, we do not find informative neither the main effects, nor most of the interactions, as they try to find a difference between Alpha and Theta power in different locations and conditions. True enough, Alpha and Theta power were hypothesized to go in different directions, but we have no interest in them being significantly different to one another, as Alpha can change/not change in any direction regardless of any effect in Theta, which would still be meaningful (as it is the case). Therefore, we think that the approach we used (direction specific t-test) is the most correct one.

Nonetheless, as mentioned before, we report the results of the 2x2x2 ANOVA so the Reviewer can check them.

| **Term** | **p-value** |
| --- | --- |
| Condition | 0.191 |
| Band | 3.93e-8 |
| Location | 0.00369 |
| Condition * Band | 0.133 |
| Band * Location | 3.09e-6 |
| Condition * Location | 0.427 |
| Condition * Band * Location | 0.823 |

**Please better describe the item by item correlation analyses.**

**RESPONSE 20:** We have better described the correlation analysis (see page 10).

**Figure 2 is incomplete: in the time scale please replace s with ms, add the time values of the window used for the analyses and the time values of the window that resulted significant in the comparison between the two conditions. Add a plot showing the theta power for control condition**

**RESPONSE 21:** We thank the Reviewer for this comment. Figure 2 has been remade.

**Fig 4: what does proportion of correct responses (y-axis) mean, how it is calculated?**

**RESPONSE 22:** Figure 4 links performance in the memory test (accuracy in the y-axis) and the Theta power (x-axis) for each image used in the study. Accuracy for each image is the proportion of correct recognition responses (yes) across participants for that image in the memory test (N=27).
